# Development of Macular Holes after Rhegmatogenous Retinal Detachment Repair in Japanese Patients

**DOI:** 10.1155/2012/740591

**Published:** 2012-01-18

**Authors:** Mamiko Shibata, Toshiyuki Oshitari, Fusae Kajita, Takayuki Baba, Eiju Sato, Shuichi Yamamoto

**Affiliations:** Department of Ophthalmology and Visual Science, Chiba University Graduate School of Medicine, Inohana 1-8-1, Chuo-ku, Chiba 260-8670, Japan

## Abstract

*Purpose*. To determine the factors associated with the development of a macular hole (MH) after successful rhegmatogenous retinal detachment (RRD) surgery. *Methods*. Of 1260 eyes that underwent surgery for RRD between April 2005 and March 2010 in our hospital, the medical records of 4 cases from our hospital and one case from another hospital that had undergone RRD surgery and later developed MH were reviewed. This is a retrospective study. *Results*. 837 eyes underwent pars plana vitrectomy (PPV) with or without scleral buckling (SB), and 423 eyes underwent SB. The four cases that developed MH had PPV alone and one case had PPV with SB. After including the results of three earlier reports, the mean interval for the MH to develop after SB alone was significantly shorter than after PPV alone or after PPV with SB. *Conclusions*. The SB procedures might accelerate the development of MH after RRD surgery.

## 1. Introduction

Idiopathic macular holes (MHs) develop from tangential anteroposterior traction by the vitreous [1, 2]. This has been supported by histopathological studies and optical coherence tomographic (OCT) images [3, 4]. However even after pars plana vitrectomy (PPV) with removal of all tractions, MHs can develop in several diseases [5]. Thus, the development of a MH appears to be independent of a vitreofoveal traction in some cases. Lipham and Smiddy suggested that cystic degeneration accompanied by glial proliferation along the margins of the MHs may be associated with the formation of an atypical macular shape after PPV [5]. However, it should take long time to develop full-thickness MH by the traction of glial plaques and membrane formation. In fact, Sheth and Bainbridge reported the cases of the delayed development of MH after PPV for rhegmatogenous retinal detachment (RRD) [6]. They explain that the delayed onset may cause gradual retinal glial tissue contraction surrounding the full-thickness MH [6].

 Previous studies indicate that a MH can develop after rhegmatogenous retinal detachment (RRD) repair by scleral buckling (SB) [7, 8]. The development of a MH after RRD repair has been found after PPV with or without concomitant SB [6, 9–11]. We noticed that there appeared to be a difference in the time for the MH to develop after RRD repair among the eyes that had undergone SB, PPV, or PPV with SB. We hypothesized that the time at which MH develops after RRD surgery is an important factor that is related to the pathogenesis of a MH.

 We reviewed our surgical records for the past 6 years and determined the incidence of MH development after RRD repair in our hospital. In addition, we reviewed the records of patients who developed a MH after RRD surgery in three articles published from 2008 and combined these data with ours [6, 10, 11]. These patients were analyzed by the type of RRD surgery they had undergone: SB alone, PPV alone, or PPV with SB [6, 10, 11]. Statistical analyses were performed on the mean intervals to develop a MH after RRD repair to determine the pathogenesis of the MH development after RRD surgery.

## 2. Methods

We examined the medical records of all patients who had undergone surgery for a RRD from April 2005 to March 2010 at the Chiba University Hospital. The types of surgical procedure for RRD repair were SB, PPV, or PPV + SB. Pneumatic retinopexy for RRD surgery was not performed in our hospital. During performing PPV for RRD surgery in our hospital, any remaining vitreous was meticulously removed by using triamcinolone acetonide to visualize residual cortical vitreous.

 This is a retrospective study, and we frame our findings within the context of the previous published papers [6, 10, 11]. All patients were informed of surgical procedures, and they provided written consent forms. All procedures used conformed to the tenets of the World Medical Association Declaration of Helsinki. Approval was obtained from the Institutional Review Board of Chiba University Hospital to perform this study.

 We found four patients had developed a MH after the RRD repair (0.32%). We also examined the records of one patient who was referred to our hospital because of a MH that was developed after RRD surgery. The age, gender, visual acuity at the time of MH development, the type of RRD surgery, the interval between the RRD surgery and the development of a MH, foveal involvement of the RD, final visual acuity after MH repair, and the results of the MH surgery were recorded. The status of the macula was determined by OCT (OCT3000 and/or spectral domain OCT) before and after the MH surgery.

 All of our patients with a MH underwent PPV with removal of the internal limiting membrane (ILM) and intraocular gas tamponade.

 We also reviewed three reports published since 2008 in Europe and Japan on eyes that had developed a MH after RRD repair [6, 10, 11]. Because the report published in 1999 did not describe the clinical information of the patients who developed a MH after RRD repair, we did not include their data for the analyses [9].

 We separated all patients including ours into the types of RD surgery: SB alone, PPV alone, and PPV with SB. Because pneumatic retinopexy for RRD surgery was not commonly performed in Japan, we did not include the single patient who underwent pneumatic retinopexy in the Benzerroug's et al. report [10].

 The clinical data were statistically analyzed by student *t* tests, paired *t* tests, or chi-square tests. A *P* < 0.05 was considered significant. Regarding the mean intervals for the MH development after SB, PPV with SB, and PPV, parametric comparisons were used for the analysis of variance (ANOVA). If ANOVA was significant, the significance of individual differences was evaluated by the Bonferroni/Dunn test. The data are expressed as the mean ± standard deviation (SD), and the Stat View 5.0 program was used for all statistical analyses.

## 3. Results

Between April 2005 and March 2010, 1260 eyes underwent surgery for RRD; 837 eyes underwent PPV with or without SB, and 423 eyes underwent SB alone. One case that had developed a MH after PPV for a RRD at another hospital but was treated at our hospital for the MH was also studied. Four eyes that underwent the RRD surgery in our hospital developed a full-thickness MH (0.32%). Overall, there were three men and two women with a mean age of 49 years (range 16 to 60 years) who developed a MH after PPV with or without SB. Posterior vitreous detachment (PVD) was identified in three cases (patients 1, 2, and 4) at the time of RD diagnosis, and two cases (patients 2 and 4) had high myopia. Thus, PVD did not protect against MH development and high myopia was not an essential factor for development of MH after RRD surgeries. The mean interval between the RRD surgery and onset of a MH was 25 months with a range from 0.27 to 65 months. In all cases, the MH was closed by PPV with internal limiting membrane (ILM) removal and intraocular gas tamponade (100%). The clinical data of all cases are shown in Table 1. The visual acuity of Patient number 1 was not measured at the time of the MH diagnosis because the interval was too short.

**Figure 1:**
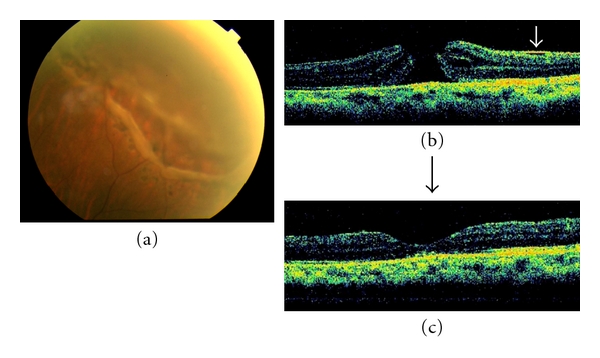
Fundus photographs and optical coherence tomographic (OCT) images of a representative patient (patient 4). (a) Fundus photograph shows a retinal break surrounding the scar of photocoagulation with retinal detachment. (b) OCT image showing a full-thickness MH after RRD repair. A white arrow showed a secondary epiretinal membrane. (c) The MH is closed by PPV with ILM removal and gas tamponade. The final VA was 0.2.

**Figure 2:**
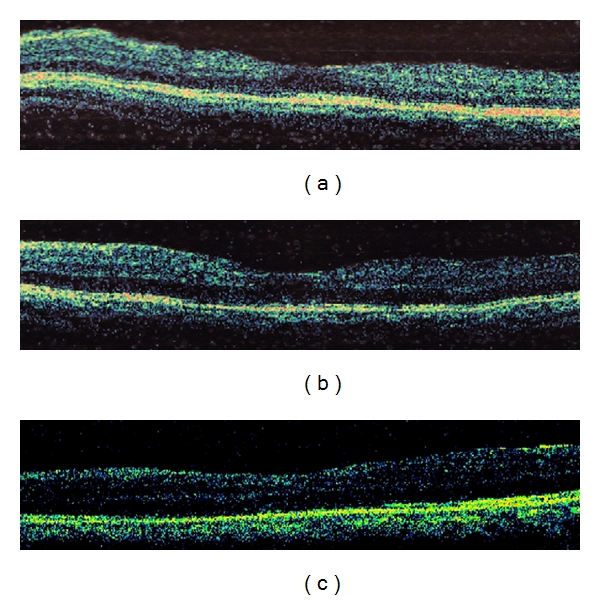
OCT images of Patient 5 before and after RD repair to see macular status before MH development. (a) The OCT image before the first RRD surgery. (b) The OCT image after the first RRD surgery (SB). (c) The OCT image after the second RRD surgery (PPV 10 months after the first surgery). MH was not developed yet. Four years later, she underwent the third operation for RRD repair (PPV + SB).

 Fundus photographs and the OCT images of a representative case (Patient 4) are shown in Figure 1. OCT images before and after RD repair (Patient 5) to see macular status before MH development are shown in Figure 2.

We summarize all of the published data from 2008 including ours on eyes that developed a MH after RRD surgery in Table 2 [6, 10, 11]. There was no significant difference in the ages among the SB-alone, PPV-alone, and PPV- with SB-groups. In the SB group, the number of macular off RD was significantly higher than that in the PPV-alone group. The visual acuities were significantly improved after the MH closure in the SB group and the PPV- with SB-group. The mean interval for the MH development after SB alone was significantly shorter than that after PPV with SB (Student's *t*-test) or after PPV alone (Student's *t*-test, the Bonferroni/Dunn test) (Table 2).

## 4. Discussion

The development of a MH after RRD repair is a rare occurrence. The prevalence of a development of a MH after RRD repair has been reported to be from 0.5 to 2.0% [8–11], but the incidence was less in our clinic. In the USA and Europe, a MH develop most often in eyes that had undergone SB for the RRD [7, 8, 10, 12]. However, none of our 423 cases that underwent SB surgery alone developed a MH. We could not determine whether the absence of MH development after SB was related to the ethnicity. Additional multicenter-based studies are required to answer this question.

 From the combined results, all of the cases that developed a MH after SB had the macular off type of RRD. The macular-off type of RD may be due to a greater anteroposterior vitreofoveal traction that causes the RD. However, in the PPV-alone and the PPV + SB groups, the macular status may not be related to the development of a MH.

 The main focus of this study was the differences in the interval between the development of a MH and the RRD surgery for the SB-alone, PPV-alone, and PPV + SB groups. The interval was significantly shorter in the SB group than in the PPV + SB or the PPV groups. In the PPV + SB group, the interval was shorter than the PPV group although the difference was not significant. We recognize that the group of the patients included in the statistical analysis is small and heterogeneous. Thus, the results of this analysis should be interpreted with caution.

 We can make three tentative conclusions from these findings. First, the vitreous and anteroposterior vitreomacular traction is not essential for the development of a MH after RRD repair. Second, the existence of the vitreous during the SB surgery may facilitate the development of a MH after RRD repair. Third, the results support the hypothesis that glial migration and proliferation followed by the contraction of glial plaques or secondary membrane formation surrounding the MH is associated with the development of a MH after PPV [5]. Surgical procedures to remove the ILM can eliminate the glial plaques or secondary membrane formation. The successful results of our surgeries support these conclusions. The formation of a MH by the contraction of glial plaques or secondary membrane formation probably requires a longer time without vitreomacular traction. During this period, the atrophic changes of macula may proceed. Thus even after closure of a MH, the final visual acuities may not significantly improve in the PPV group.

## 5. Conclusion

The development of a MH after RRD repair is very rare with an incidence of 0.32% in our population. The MHs can be closed completely by PPV with ILM removal and a gas tamponade. Although it is a circumstantial evidence, the existence of vitreous and anteroposterior vitreofoveal traction is not required for the development of a MH, and SB and the existence of vitreous may facilitate the MH development after RRD repair.

## Figures and Tables

**Table 1:** Patient's information.

Patient number	Age at MH	Sex	Fovea On/off	Type of RD repair	Time to MH (months)	VA at MH diagnosis	Final VA	Hole closure
No. 1	56	F	Off	PPV	0.27	NA	0.15	Closed
No. 2	53	M	On	PPV	0.54	0.09	0.05	Closed
No. 3	60	M	NA	PPV	52	0.5	0.7	Closed
No. 4	60	M	On	PPV	65	0.2	0.2	Closed
No. 5	16	F	Off	1st, SB 2nd, PPV 3rd, PPV + SB	7	0.15	0.2	Closed

MH: macular hole, M: male, F: female, NA: not available, RD: retinal detachment, VA: visual acuity, PPV: pars plana vitrectomy, SB: scleral buckling.

**Table 2:** Differences among the types of RD surgeries: SB, PPV + SB, and PPV.

	SB (*n* = 7)	SB + PPV (*n* = 5)	PPV (*n* = 6)
Age (years)	61 ± 4	46 ± 19	60 ± 6
*Gender	Men 5, women 2	Men 1, women 4	Men 5, women 1
**Macular status	On 0, off 7	On 2, off 3	On 3, off 2, NA 1
VA at MH (logMAR)	1.09 ± 0.23	0.84 ± 0.34	0.88 ± 0.57
***Final VA	0.76 ± 0.31	0.66 ± 0.38	0.83 ± 0.63
****Diagnostic delay (months)	1.0 ± 0.9	10.6 ± 11.1	29.6 ± 26.6

There was no significant difference in the ages among the three groups (*t* test). *In the SB + PPV group, there were significantly more women than in the PPV group (chi-square test). **In the SB group, macular off was significantly increased compared to the PPV group (*t*-test). ***Final VAs were significantly improved compared to the SB and PPV + SB groups (paired *t*-test). ****The mean times of diagnostic delay were significantly longer in PPV + SB and PPV than SB (*t*-test). SB: scleral buckling, PPV: pars plana vitrectomy, VA: visual acuity, RD: retinal detachment.
